# Development and Validation of a Bioanalytical LC-MS/MS Method for Simultaneous Determination of Sirolimus in Porcine Whole Blood and Lung Tissue and Pharmacokinetic Application with Coronary Stents

**DOI:** 10.3390/molecules26020425

**Published:** 2021-01-15

**Authors:** Thi-Thao-Linh Nguyen, Van-An Duong, Dang-Khoa Vo, Jeongae Jo, Han-Joo Maeng

**Affiliations:** 1 College of Pharmacy, Gachon University, Incheon 21936, Korea; linh.nguyen@gachon.ac.kr (T.-T.-L.N.); anduong@gachon.ac.kr (V.-A.D.); vodangkhoa135@gmail.com (D.-K.V.); 2 Department of New Drug Development, Inha University College of Medicine, Incheon 22212, Korea; jeae1028@gmail.com

**Keywords:** sirolimus, LC-MS/MS, validation, whole blood, lung tissue, pharmacokinetic, bio-distribution

## Abstract

Sirolimus is a hydrophobic macrolide compound that has been used for long-term immunosuppressive therapy, prevention of restenosis, and treatment of lymphangioleiomyomatosis. In this study, a simple and sensitive liquid chromatography-tandem mass spectrometry (LC-MS/MS) was developed and validated for the simultaneous determination of sirolimus in both porcine whole blood and lung tissue. Blood and lung tissue homogenates were deproteinized with acetonitrile and injected into the LC-MS/MS system for analysis using the positive electrospray ionization mode. The drug was separated on a C18 reversed phase column with a gradient mobile phase (ammonium formate buffer (5 mM) with 0.1% formic acid and acetonitrile) at 0.2 mL/min. The selected reaction monitoring transitions of *m*/*z* 931.5 → 864.4 and *m/z* 809.5 → 756.5 were applied for sirolimus and ascomycin (the internal standard, IS), respectively. The method was selective and linear over a concentration range of 0.5–50 ng/mL. The method was validated for sensitivity, accuracy, precision, extraction recovery, matrix effect, and stability in porcine whole blood and lung tissue homogenates, and all values were within acceptable ranges. The method was applied to a pharmacokinetic study to quantitate sirolimus levels in porcine blood and its distribution in lung tissue following the application of stents in the porcine coronary arteries. It enabled the quantification of sirolimus concentration until 2 and 14 days in blood and in lung tissue, respectively. This method would be appropriate for both routine porcine pharmacokinetic and bio-distribution studies of sirolimus formulations.

## 1. Introduction

Sirolimus (also known as rapamycin) is a hydrophobic macrolide compound produced by a strain of the bacterium *Streptomyces hygroscopicus* in Easter Island [1]. It was first isolated and developed as an antifungal drug with potent anticandidal activity [2]. However, later studies revealed that sirolimus possessed intense antitumor, immunosuppressive, and antiproliferative activities due to its ability to bind and inhibit the mechanistic target of rapamycin (mTOR) [3,4]. Sirolimus interacts and forms a complex with the intracellular immunophilin FK binding proteins 12, which blocks mTOR. This inhibition causes the suppression of cytokine driven T-lymphocyte proliferation, and consequently stops the progression of cell-cycle from the G1 to the S phase [5]. In terms of pharmacokinetic properties, sirolimus in solution is rapidly absorbed from the human gastrointestinal tract and reaches the maximal blood concentration after 2 h [6]. It distributes extensively into red blood cells (95%) [7].

Sirolimus (Rapamune^®^, Pfizer) was approved by the United States Food and Drug Administration (US FDA) in 1999 and is currently used for long-term immunosuppressive therapy in solid organ transplantation [5,8,9]. Additionally, a sirolimus-eluting coronary stent has been developed, which demonstrated remarkable efficacy in eliminating the occurrence of restenosis due to the antiproliferative effect of sirolimus. The stent was approved by the US FDA in 2003 (Cypher^®^, Johnson & Johnson) [10]. In 2015, sirolimus became the first drug approved by the US FDA for the treatment of lymphangioleiomyomatosis (LAM), a rare, progressive, cystic lung disease associated with inappropriate activation of mTOR signaling [11,12,13]. In addition, sirolimus is a potential treatment therapy for lupus [14], lymphatic malformation [15], skin cancer [16], and lung cancer [17].

Sirolimus has a narrow therapeutic window. Its adverse effects correlate with its concentration in the blood, and thus, therapeutic drug monitoring of sirolimus in whole blood is required [18,19]. To determine the level of sirolimus, microparticle enzyme immunoassay, liquid chromatography with UV-Vis detection, and liquid chromatography-tandem mass spectrometry (LC-MS/MS) are used. LC-MS/MS has been widely used for drug quantification and pharmacokinetics studies with the advantages of high selectivity and sensitivity. It allows the determination of the targeted drug independently from its metabolites in one short run. Various LC-MS/MS methods have been developed and validated for therapeutic drug monitoring of sirolimus [20]. Some methods allowed the simultaneous determination of sirolimus and other drugs, including everolimus, cyclosporine A, and tacrolimus [21,22,23,24]. Several studies presented fast, robust, and straightforward LC-MS/MS methods that have been used for the analysis of clinical samples in daily routine (70–120 samples/day) [25,26]. In addition, some methods required a stable isotope internal standard (^13^C_2_D_4_-everolimus) [27]. Several studies developed LC-MS/MS methods for quantification of sirolimus on dried blood spots, allowing the patient to sample at home and save patients’ transportation costs and time [28,29]. Further, some LC-MS/MS methods were developed and validated to determine sirolimus in laboratory animal samples, such as dog blood [30] and rabbit aqueous humor [31]. However, to the best of our knowledge, among several developed LC-MS/MS methods, few have yet to be used for the analysis of sirolimus in tissues [32]. Determination of drug levels in tissues is essential for studying the tissue distribution of the drug. In the present study, a fast, simple, and reliable LC-MS/MS method for the determination of sirolimus in both porcine whole blood and lung tissue was developed and fully validated. This method was successfully applied to the pharmacokinetic and bio-distribution studies of a sirolimus-eluting stent in porcine coronary arteries.

## 2. Results and Discussion

### 2.1. Method Development

**Figure 1:**
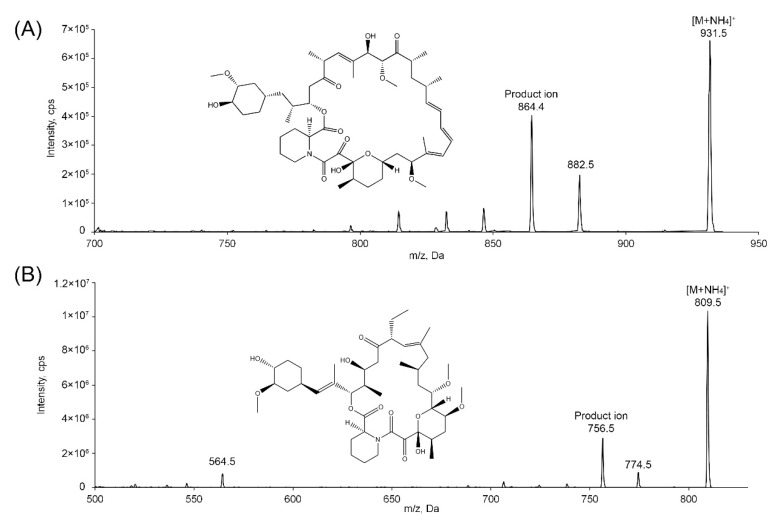
Chemical structures and product ion scan spectra of (**A**) sirolimus and (**B**) ascomycin (IS) in positive ionization mode. Working parameters were similar to those in Table 1, except for declustering potential (130 V), collision energy (50 V), and collision cell exit potential (0 V).

The chemical structures of sirolimus and ascomycin (internal standard, IS) are depicted in Figure 1. With the positive ionization mode, sirolimus and IS exhibited ammonium adducts [M + NH_4_]^+^ at *m*/*z* 931.5 and 809.5, respectively, which are frequently observed in ammonia-mediated ionization mass spectra of compounds with oxygen groups (ethers, aldehydes, ketones, and esters) in their structures [33]. In the product ion spectra (Figure 1), the most prominent fragment ions were at *m*/*z* 864.4 for sirolimus and *m*/*z* 756.5 for IS, which is in accordance with previous findings [21,34]. The product ion at *m*/*z* 864.4 of sirolimus was likely formed due to the separation of an -OCH_3_ and two H_2_O, whereas the product ion at *m*/*z* 756.5 of the IS was potentially the result of three H_2_O separation. The declustering potential, collision energy, and collision cell exit potential were optimized to obtain maximum responses of sirolimus and the IS (Table 1). Thereafter, the selected reaction monitoring (SRM) quantitative analyses were performed with *m*/*z* 931.5 → 864.4 for sirolimus and *m*/*z* 809.5 → 756.5 for the IS. An ammonium formate solution (5 mM) was employed as a mobile phase component to induce the ammonium adducts of sirolimus and the IS. Several mobile phase compositions and reverse phase columns were tested to produce peaks of sirolimus and the IS with adequate retention time and separation. Peaks of sirolimus and IS were not appropriately observed with isocratic modes; thus, gradient modes were used. Finally, a mixture of ammonium formate buffer (5 mM) with 0.1% formic acid (A) and acetonitrile (B) in gradient mode (0–1 min: 20% B, 3–6 min: 80% B, 6.1–8.5 min: 20% B) and a Synergi^TM^ 4 μm polar-RP 80A column were used to obtain optimized peaks of sirolimus and the IS.

### 2.2. Method Validation

#### 2.2.1. Selectivity

**Figure 2:**
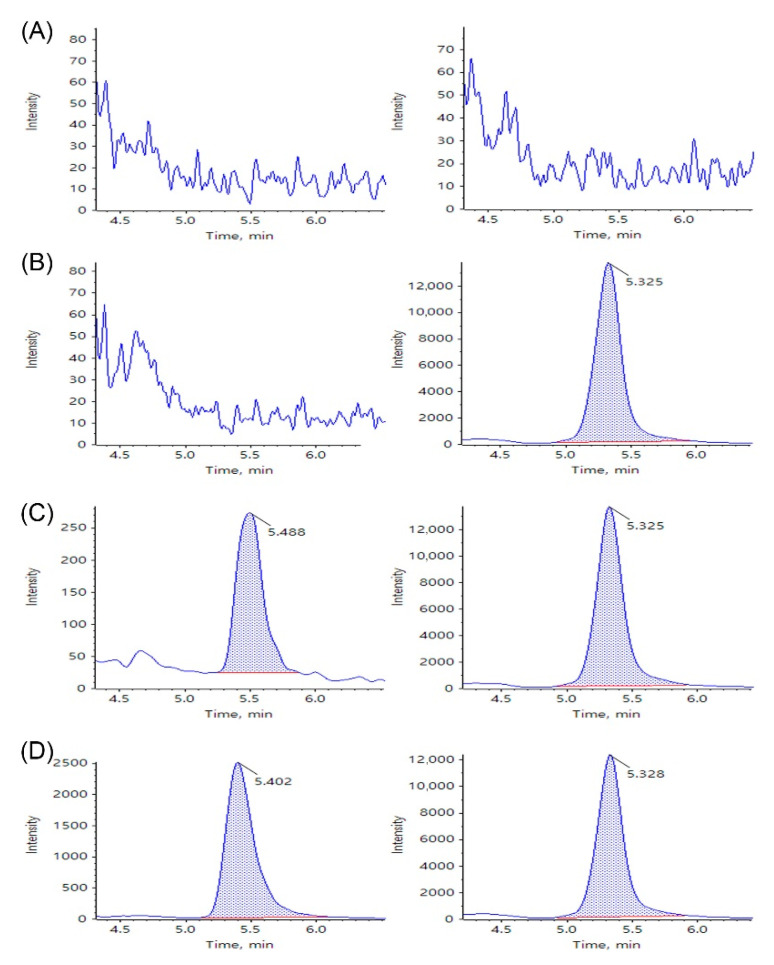
Selected reaction monitoring (SRM) liquid chromatography-tandem mass spectrometry (LC-MS/MS) chromatograms of sirolimus (**left**) and IS (**right**) obtained by deproteinization of (**A**) blank porcine whole blood, (**B**) blank porcine whole blood spiked with 1 ng/mL of IS, (**C**) blank porcine whole blood spiked with sirolimus at lower limit of quantification (LLOQ) 0.5 ng/mL and 1 ng/mL of IS, and (**D**) a porcine whole blood sample at 5 min in the pharmacokinetic studies.

**Figure 3:**
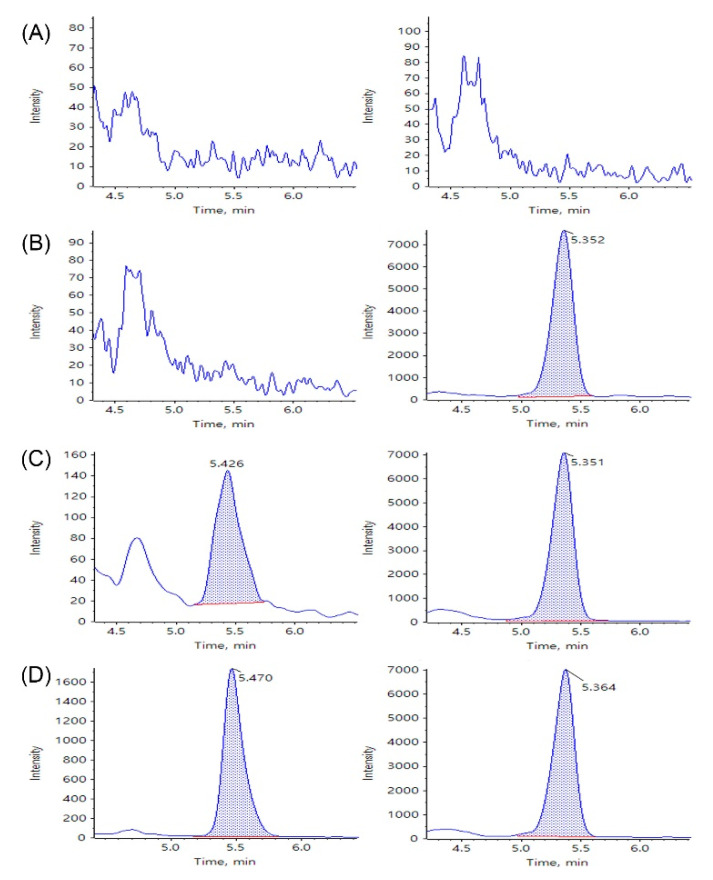
Selected reaction monitoring (SRM) LC-MS/MS chromatograms of sirolimus (**left**) and IS (**right**) obtained by deproteinization of (**A**) blank porcine lung tissue, (**B**) blank porcine lung tissue spiked with 1 ng/mL of IS, (**C**) blank porcine lung tissue spiked with sirolimus at LLOQ 0.5 ng/mL and 1 ng/mL of IS, and (**D**) a porcine lung sample at 1 h in the pharmacokinetic studies.

Typical chromatograms of blank porcine whole blood/lung tissue, zero calibrators, a sirolimus standard, and a porcine sample from the pharmacokinetic studies are shown in Figure 2 and Figure 3. Peak area and retention time of sirolimus and IS in these samples are presented in Tables S1 and S2 (Supplemental Information). No interference at the retention times of sirolimus or IS was observed in the chromatograms of the blank porcine whole blood and lung tissue samples (Figure 2A and Figure 3A). In the zero calibrators, there are only peaks of the IS (Figure 2B and Figure 3B). Symmetrical peaks of sirolimus and the IS were obtained with adequate retention times of approximately 5.4 and 5.3 min, respectively (Figure 2C,D and Figure 3C,D). Moreover, porcine whole blood/lung tissue samples from pharmacokinetic studies showed no difference in retention times of sirolimus and the IS compared with those of the standard whole blood/lung tissue samples. These observations suggest that the selectivity of the analysis assay for sirolimus was adequate.

#### 2.2.2. Linearity

Under the developed analytical conditions, the calibration curves for sirolimus in porcine whole blood were linear over a concentration range of 0.5–50 ng/mL. The calibration equation for sirolimus was y = (0.05227 ± 0.00373) x + (0.00766 ± 0.00251), with R^2^ = 0.99317 ± 0.00150 (*n* = 5, means ± SDs, using the weighted (1/x) least squares regression analysis), where y, x, and R refer to peak area ratio (sirolimus/IS), sirolimus concentration in whole blood, and the correlation coefficient, respectively. The calibration curves (peak area ratio versus concentration) for sirolimus in porcine lung tissue were linear over a range of 0.5–50 ng/mL with the developed analytical conditions. Using the weighted (1/x) least squares regression analysis, the calibration equation for sirolimus was y = (0.05056 ± 0.00356) x + (0.00003 ± 0.00553), with R^2^ = 0.99792 ± 0.00162 (*n* = 5, means ± SDs), where y, x, and R refer to peak area ratio (sirolimus/IS), sirolimus concentration in lung tissue, and the correlation coefficient, respectively. The weighted (1/x) least squares regression analysis demonstrated the acceptable precision and accuracy of the corresponding calculated concentrations at each level for both whole blood and lung tissue. The precision was 4.33–13.26% for whole blood and 1.66–10.52% for lung tissue, whereas the accuracy was 90.42–107.63% for whole blood and 94.06–102.29% for lung tissue. Thus, the response (sirolimus/IS peak ratio) was directly proportional to the whole blood/lung tissue concentration of sirolimus, indicating the linearity of the LC-MS/MS assay in the range of 0.5–50 ng/mL.

#### 2.2.3. Sensitivity

The lower limit of quantification (LLOQ) for the LC-MS/MS assay was established at a concentration of 0.5 ng/mL. In porcine whole blood, the LLOQ showed an accuracy of 95.25% and a precision of 14.14%, whereas, in porcine lung tissue, the LLOQ showed an accuracy of 110.79% and a precision of 1.85% (Table 2). The signal-to-noise for samples was ≥10 at this concentration. Hence, the LLOQ response satisfied the acceptance criteria with an accuracy in the range 80–120% and a precision of ≤20% for both types of samples [35]. The limit of detection (LOD) was 0.15 ng/mL and 0.3 ng/mL for whole blood and lung tissue samples, respectively, as calculated with the equation LOD = 3.3 σ/m. In addition, similar values of LOD were obtained when using the signal-to-noise ratio criterion of 3:1 in experimental determination. In a previous report, LLOQ was 2.5 mg/L in human blood [34]. Another LC-MS/MS method using dog blood showed a lower LLOQ (0.2 ng/mL) but a narrower linear range (0.2–16 ng/mL) [30].

#### 2.2.4. Accuracy and Precision

Quality control (QC) samples were analyzed in five replicates over five different days to determine the intra-day and inter-day accuracy/precision. Four levels of sirolimus in porcine whole blood/lung tissue were investigated, including LLOQ (0.5 ng/mL), low QC (LQ, 1.5 ng/mL), middle QC (MQ, 15 ng/mL), and high QC (HQ, 40 ng/mL). Table 2 shows a summary of the intra-day and inter-day accuracy/precision for porcine whole blood and lung tissue samples. In detail, the intra-day accuracy of sirolimus was in the range of 94.20–95.92% for whole blood and 97.34–110.79% for lung tissue samples (relative error, RE). The intra-day precision was ≤14.14% for whole blood and ≤2.93% for lung tissue samples (coefficient of variation, CV). The inter-day accuracy of sirolimus in whole blood and lung tissue samples was 94.83–101.30% and 92.97–102.91%, respectively, whereas the inter-day precision was ≤10.48% and ≤10.06%, respectively. Thus, the accuracy and precision of the LC-MS/MS assay for sirolimus determination in porcine whole blood and lung tissue were within the acceptable limits recommended by the bioanalytical method validation guideline of the US FDA in 2018 [35].

#### 2.2.5. Extraction Recovery and Matrix Effects

The extraction recovery was evaluated by comparing the peak responses of sirolimus QC samples and the IS with those of blanks spiked with the analytes after extraction at the same concentrations. The extraction recovery values were similar for all levels of QC within the same type of sample matrix (Table 3). They ranged from 56.80% to 63.33% for whole blood and 80.00–82.91% for lung tissue samples. The extraction recovery was 102.43% and 83.00% for the IS in whole blood and lung tissue, respectively.

The matrix effect was investigated in three different porcine lung tissue samples (Table 4). The peak responses of QC samples and the IS prepared with extracted blank tissue (set 1) were compared with those of the standard solutions at the same concentrations (set 2) to calculate the absolute matrix effect. The absolute matrix effect values in porcine whole blood samples were 104.84–107.53% for sirolimus and 93.28% for the IS, whereas, in the lung tissue, those values were approximately 107% and 106%, respectively.

The relative matrix effect (CV, %) was assessed by a direct comparison of the peak areas among samples in the same QC level (set 1). For porcine whole blood samples, the precision of set 1 was in the range 0.77–8.43%, which was comparable with that of set 2. For lung tissue samples, the precision ranged from 1.15% to 6.91%, slightly narrower than the values from set 2. These results indicate that there was no significant effect of the matrix for the analysis of sirolimus and IS in porcine whole blood and lung tissue samples using the developed LC-MS/MS method.

#### 2.2.6. Stability

At short-term storage conditions (4 h, at room temperature ~25 °C), stock solutions of sirolimus (50 ng/mL) and the IS (1 ng/mL) showed the stability of 100.80% and 101.58% compared to fresh-prepared solutions, respectively. After long-term storage at −80 °C for 3 months, the stability was 101.26% for sirolimus and 95.91% for the IS. These data indicate that sirolimus and the IS are stable in the stock solution during both short- and long-term storage.

The stability of sirolimus was investigated in porcine whole blood and lung tissue at three different QC (LQ, MQ, and HQ) levels over various typical handling and storage conditions (Table 5). The post-preparative stability of samples stored in an autosampler (24 h, 4 °C) showed negligible changes in the peak area ratios of sirolimus and the IS (95.63–107.46% for whole blood and 99.27–101.13% for lung tissue samples). After three freeze–thaw cycles, changes in peak ratios for whole blood and lung tissue samples were within the acceptable range for stability (85.41–92.92% and 105.75–106.51%, respectively). With regard to the short-term stability, peak area ratios were similar to those of fresh-prepared whole blood and lung tissue samples (91.19–98.48% and 92.44–96.67%, respectively). Sirolimus was stable in samples after long-term storage at −80 °C, with a stability of 94.62–100.61% in whole blood and 101.01–106.24% in lung tissue samples. Collectively, sirolimus is stable in the investigated matrix (porcine whole blood and lung tissue) during typical experimental handling and storage conditions.

#### 2.2.7. Carry-Over

Carry-over study was conducted to measure residual analyte from a preceding sample that remains in the analytical instrument. Obtained results for carry-over of sirolimus and IS are shown in Table S3, Figure S1 (for blood samples), Table S4 and Figure S2 (for lung tissue samples). As evidenced, no peak of sirolimus and IS were detected in blank samples analyzed after standard samples at 50 ng/mL (upper limit of quantitation, ULOQ), indicating no potential carry-over of sirolimus and IS that can affect accuracy and precision of the assay.

### 2.3. Applicability to Pharmacokinetic and Bio-Distribution Studies

**Figure 4:**
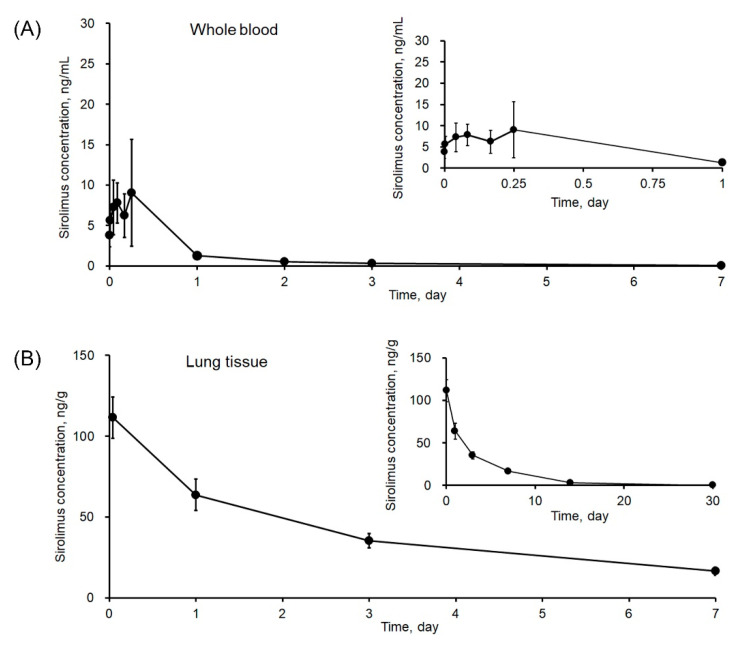
Sirolimus concentration–time profiles in (**A**) porcine whole blood (*n* = 12–24) and (**B**) lung tissue (*n* = 3) following the application of a stent device containing sirolimus. The insets show sirolimus concentration–time profiles within 1 day for whole blood and 30 days for lung tissue.

The method development and validation, as well as the pharmacokinetic studies, were conducted using pigs, since it has been used as an animal model to evaluate sirolimus-eluting stent in coronary arteries [36]. The validated bioanalytical method was used to determine the concentration of sirolimus in porcine whole blood and lung tissue following the application of stent devices containing sirolimus. The whole blood/lung tissue sirolimus concentration–time profiles are shown in Figure 4. The number of lung tissue samples at each time point was three. However, because the animals were sacrificed at 0 h, 1 h, 6 h, 1 day, 3 days, 7 days, 14 days, 30 days, and 90 days (3 animals per time point), the numbers of blood samples were different at each time point (e.g., 24 samples at 0, 5 min, and 1 h; 21 samples at 2, 4, and 6 h; 18 samples at 1 day; 15 samples at 2 and 3 days; 12 samples at 7 days). Thus, the pharmacokinetic parameters were calculated using the average concentrations (Table 6). In whole blood, the concentration of sirolimus fluctuated in the range of 3.81–9.02 ng/mL in the first 6 h, followed by rapid decreases to 1.26 ng/mL at 1 day and 0.52 ng/mL at 2 days. From day 3 onwards, its concentration was below LLOQ (i.e., 0.5 ng/mL). As a result, the maximum average concentration of sirolimus in whole blood (C_max_) was 7.79 ng/mL, and the T_max_ was 2.26 hr. The systemic exposure up to the last time point (i.e., AUC_0–48 h_) was 156.9 ng/mL·h, and the AUC_inf_ was 164.8 ng/mL·h (Table 6). The concentration of sirolimus in lung tissue samples quickly reached the maximum concentration of 111.47 ng/g (~22.29 ng/mL sirolimus in homogenate) at 1 h and decreased gradually to 2.89 ng/g (~0.58 ng/mL sirolimus in homogenate) at day 14 (Figure 4B). After this time point, its level was lower than the LLOQ. The pharmacokinetic results in our study are in agreement with a previous study, which used poly (D, L-lactide-co-glycolide) (PLGA, LA: GA = 70:30) as a polymer for the drug coating [37]. With a sirolimus dose of 151 µg/pig, the drug concentration in that study also quickly dropped to approximately 0.79 ng/mL at 1 day and was undetectable from 3 days onwards. Notably, it was reported that the drug concentration in the lung was too low to be determined, whereas, in our study, the developed method successfully enabled the determination of sirolimus in lung tissue up to 14 days. These observations suggest that the developed LC-MS/MS method would be appropriate for the determination of sirolimus levels in the preclinical pharmacokinetic and bio-distribution studies.

## 3. Materials and Methods

### 3.1. Reagents and Materials

Sirolimus (stock solution in acetonitrile, 1 mg/mL), ascomycin (stock solution in acetonitrile, 1 mg/mL), formic acid, and ammonium formate were purchased from Sigma-Aldrich (St Louis, MO, USA). HPLC-grade acetonitrile and water were supplied by Honeywell Burdick & Jackson (Muskegon, MI, USA). All other reagents were of analytical grade and were used without any further purification.

### 3.2. Instrumentation and Analytical Conditions

Sirolimus was analyzed using an LC-MS/MS system consisting of an AB SCIEX Triple Quad 5500 mass spectrometer (Applied Biosystems-SCIEX, Concord, ON, Canada) equipped with a turbo ion spray interface in positive ionization mode, an Agilent LC 1200 Binary pump system (Agilent Technologies, Santa Clara, CA, USA), and a CTC analytics autosampler (CTC Analytics AG, Zwingen, Switzerland). Ascomycin was used as an IS. A Synergi^TM^ 4 μm polar-RP 80A column (75 mm × 2.0 mm, 2.6 μm, Phenomenex, Torrance, CA, USA) equipped with a SecurityGuard^TM^ column (4.0 mm × 3.0 mm) was utilized to optimally separate sirolimus and the IS from endogenous substances of porcine whole blood and lung tissue. The mobile phase was a mixture of ammonium formate buffer (5 mM) with 0.1% formic acid (A) and acetonitrile (B). Sample separation was conducted using a flow rate of 0.2 mL/min and an 8.5-min gradient condition (0–1 min: 20% B, 3–6 min: 80% B, 6.1–8.5 min: 20% B). Temperatures of the column and the autosampler were maintained at 30 °C and 4 °C, respectively. The injection volume was 10 μL for whole blood and 5 μL for tissue samples. The SRM transitions of *m*/*z* 931.5 → 864.4 and *m/z* 809.5 → 756.5 were applied for sirolimus and IS, respectively. Mass data were acquired using Analyst software version 1.5.2 (Applied Biosystems-SCIEX, Concord, ON, Canada). Data analysis was processed using SCIEX OS offline software version 1.6 (Applied Biosystems-SCIEX, Concord, ON, Canada). The working parameters of the LC-MS/MS system are listed in Table 1.

### 3.3. Samples Preparation

#### 3.3.1. Tissue Homogenization

Blank lung tissue and pharmacokinetic experimental lung tissue samples were homogenized using the KT 30 homogenizer (Korea Process Technology, Seoul, Korea). Lung tissues were accurately weighed, and four-fold (*w*/*w*) cold phosphate-buffered saline (PBS) was added to process the homogenization. Tissues were kept in the ice-bath during the homogenization process, and tissue homogenates were stored at −20 °C until use.

#### 3.3.2. Preparation of Standards and Quality Control Samples

The stock solution of sirolimus (1 mg/mL, in acetonitrile) was appropriately diluted with acetonitrile to obtain sets of working standard solutions (5–500 ng/mL). All the stock and working standard solutions were stored at −80 °C until experimental analysis. Standard samples were prepared in whole blood by spiking 90 μL of blank porcine blood with 10 μL of each working standard solution. Similarly, standard samples in porcine lung tissues were prepared by spiking 15 μL of each working standard solution into 135 μL of blank lung tissue homogenate. The final sirolimus concentrations in blood and lung tissue homogenate were 0.5, 1, 2, 5, 10, 20, and 50 ng/mL. Extraction of the drug and IS was performed using a protein precipitation method as previously described [38,39]. To each standard sample, a 2-fold volume (200 μL for blood and 300 μL for tissue homogenate samples) of IS solution in acetonitrile (1 ng/mL) was added, followed by vortexing for 1 min for deproteinization. After centrifugation at 14,000 rpm for 15 min, 100 μL of the supernatant was collected for analysis using the devised LC-MS/MS methods. QC samples were separately prepared in blood and lung tissue homogenate using similar procedures. The QC sample included LLOQ (0.5 ng/mL), LQ (1.5 ng/mL), MQ (15 ng/mL), and HQ (40 ng/mL).

### 3.4. Assay Validation

The LC-MS/MS method for sirolimus analysis was validated according to the bioanalytical method validation guidelines of the US FDA in 2018, as previously described [35,40].

#### 3.4.1. Selectivity

Blank whole blood and lung tissue homogenates from six or three animals, respectively, were spiked with the IS only or with both sirolimus and IS. Chromatograms of blank blood/lung tissue, blood/lung tissue spiked with the IS only, and blood/lung tissue spiked with both sirolimus and IS were then compared for selectivity of the assay.

#### 3.4.2. Linearity

The linearity of the assay was assessed using the standard samples prepared with sirolimus blood and lung tissue homogenates over the concentration range of 0.5–50 ng/mL. Calibration curves were constructed using the peak area ratios of sirolimus and IS by weighted (1/x) linear regression analysis. R^2^ values of calibration curves were used to evaluate the linearity of the assay, using the criteria of ≥ 0.990. Accuracy and precision were evaluated during linearity runs using the deviation from the nominal concentration (RE%) and the CV%, respectively. Criteria of the accuracy and precision of the assay were set within ± 15%, except for the LLOQ, which were set at ± 20%.

#### 3.4.3. Sensitivity

The LOD was determined by the equation: LOD = 3.3 σ/m, where σ is the standard deviation of the intercept of the regression line, and m is the slope of the calibration curve. LOD was also experimentally determined using a signal-to-noise ratio of ≥ 3. The LLOQ, which is the lowest concentration of sirolimus that can be quantitatively determined with a precision of less than or equal to 20% and accuracy between 80% and 120%, was determined with a signal-to-noise ratio criterion of ≥ 10.

#### 3.4.4. Accuracy and Precision

QC samples at four different concentrations of 0.5 (LLOQ), 1.5 (LQ), 15 (MQ), and 40 (HQ) ng/mL were used to assess accuracy and precision. For intra-day accuracy and precision, five replicates of QC samples were analyzed within one day. The QC samples were analyzed in five replicates over five different days to examine the inter-day data. The acceptance criteria were set at below ± 15% of RE and within ± 15% of CV for accuracy and precision, respectively, except at the LLOQ, which were set at ± 20%.

#### 3.4.5. Extraction Recovery and Matrix Effect

The extraction recovery of the analyte was assessed to ensure that the sample extraction process is efficient and reproducible. Peak areas of extracted samples at LQ, MQ, and HQ concentrations with those of blanks spiked with sirolimus post-extraction were compared. The matrix effect was evaluated using three sources of blank whole blood or lung tissue homogenates matrix to determine whether the endogenous components of whole blood or lung tissue affect the ionization of sirolimus and IS. To determine the absolute matrix effect, mean peak areas of post-deproteinization samples (set 1) with those of neat solutions of compounds (set 2) in acetonitrile at equivalence concentrations were compared. The variability in peak areas from set 1, expressed as precision (CV, %), was determined and considered as the relative matrix effect.

#### 3.4.6. Stability

The stability of stock solutions of sirolimus and IS was assessed by comparing the peak response of freshly prepared acetonitrile solution with that of the solution stored at room temperature for 4 h or at −80 °C for 3 months of sirolimus (50 ng/mL) and IS (1 ng/mL). The stability of QC samples in porcine whole blood (LQ, MQ, and HQ) was investigated in terms of short- and long-term stability, post-preparative stability, and freeze–thaw stability. Short-term stability was assessed by keeping QC samples in laboratory condition (room temperature ~25 °C) for 4 h, and long-term stability was evaluated with QC samples stored at −80 °C for 3 months. The post-preparative stability of processed samples stored in an autosampler at 4 °C was evaluated after 24 h. Freeze–thaw stability was tested over three repeated cycles, whereby QC samples were frozen at −80 °C and then thawed at room temperature. Samples were considered stable at the test conditions if the intensity of stored samples differed by less than 15% from that of freshly prepared samples.

#### 3.4.7. Carry-Over

Carry-over of sirolimus and IS was assessed by analyzing blank samples after the highest sirolimus standard samples (50 ng/mL, ULOQ). Then, standard samples at LLOQ (0.5 ng/mL) were also analyzed after the blank samples. The experiment was repeated three times for both blood and lung tissue samples. Carry-over in the blank samples should not be greater than 20% of the sirolimus response at the LLOQ and 5% of the response for the IS.

### 3.5. Application to Pharmacokinetic and Bio-Distribution Studies in the Lung

In this study, a total of 27 male Defined Health Status pigs were utilized (average weight of 45 kg, 20–50 weeks old) (CRONEX, Seoul, Korea). Animals were randomly divided into nine groups following the sacrifice time point (blank, 1 h, 6 h, 1 day, 3 days, 7 days, 14 days, 30 days, and 90 days). As a form of randomization, animals were assigned to the study in order of their implantation. All animals were handled under the National Institutes of Health guideline, and Animal Care and Use Committee policies of the Yonsei University Cardiovascular Product Evaluation Center (CPEC, Seoul, Korea). 

For the scaffold implantation, anesthesia was induced with a mixture of 0.02–0.04 mg/kg of atropine, 2.0–8.0 mg/kg of azaperone, 0.1–1.0 mg/kg of xylazine, 5.0 mg/kg of alfaxalone, and 1.0–3.0 mg/kg of tramadol (via intramuscular injection), and maintained with a mixture of approximately 2% isoflurane in oxygen (via an endotracheal tube). About 200 IU/kg of heparin was administered intravenously before selective catheterization for the scaffold implantation. A total of 48 Phosline^TM^ scaffolds (Dotter Inc., Incheon, Korea, 3 mm × 18 mm) were implanted in 24 animals, two each. Three animals were used as blank controls. The scaffold is coated with a single layer of poly (d, l-lactic acid) (PDLLA), sirolimus (0.9 μg/mm^2^), and phosphorylcholine polymer. The total amount of sirolimus carried by the two Phosline^TM^ scaffolds per animal is 326 μg.

Implantation was performed in two of the coronary arteries (left circumflex artery, left anterior descending artery, and/or right coronary artery) per animal, as the anatomy allowed. The interventionalist decided on the suitability of the vessels, based on angiographic images of the native vessels. The scaffold was introduced into the artery by advancing the scaffolded balloon catheter through the guide catheter and over the guidewire to the deployment site. Four radiopaque markers at each end of the scaffold facilitated precise placement. After implantation, animals received 100 mg of aspirin and 75 mg of clopidogrel daily until termination.

Blood samples were obtained at pre-implantation, post-implantation, and at other time points depending on each group until sacrifice (5 min, 1, 2, 4, 6 h, 1, 2, 3, 7, 14, 30, and 90 d). Thus, the numbers of blood samples were as follows: *n* = 24 at 0, 5 min, and 1 h; *n* = 21 at 2, 4, and 6 h; *n* = 18 at 1 d; *n* = 15 at 2 and 3 d; and *n* = 12 at 7 d. Then, the blood samples were treated with ethylenediaminetetraacetic acid (EDTA). After sacrifice, lung tissues were explanted and washed with PBS. All tissues and whole blood samples were stored at −80 °C until use.

## 4. Conclusions

In this study, an LC-MS/MS bioanalytical assay method was successfully developed and validated for the determination of sirolimus levels in both porcine whole blood and lung tissue. The sample preparation required a simple protein precipitation step for sirolimus extraction. This method exhibited adequate selectivity, linearity, sensitivity, accuracy, and precision. The drug and IS were stable during typical handling and processing conditions. The method was successfully applied for pharmacokinetic and bio-distribution studies of sirolimus following the application of stents in porcine coronary arteries. To the best of our knowledge, this is the first report describing the development and validation of an LC–MS/MS assay of sirolimus in both porcine whole blood and lung tissues. This method can be applied in pharmacokinetic and bio-distribution studies to quantitate sirolimus levels in blood, as well as its distribution to various organs.

## Figures and Tables

**Table 1:** The LC–MS/MS working parameters.

Source Parameters	Sirolimus	IS
Declustering potential (V)	36	86
Entrance potential (V)	10	10
Collision energy (V)	23	29
Collision cell exit potential (V)	24	32
Collision gas (°C)	9	9
Ionspray voltage (V)	5500	5500
Temperature of ion source (°C)	500	500
Nebulizing gas (GS1) (°C)	50	50
Drying gas (GS2) (°C)	50	50
Curtain gas (°C)	50	50

**Table 2:** Accuracy and precision of sirolimus in porcine whole blood and lung tissue samples.

Nominal Conc. (ng/mL)	Whole Blood			Lung Tissue		
	Calc. Conc.	Accuracy (%)	Precision (CV, %)	Calc. Conc.	Accuracy (%)	Precision (CV, %)
Intra-day (*n* = 5)						
0.5	0.48	95.25	14.14	0.55	110.79	1.85
1.5	1.41	94.20	8.65	1.46	97.34	2.93
15	14.39	95.92	5.14	15.25	101.69	2.68
40	38.23	95.56	4.04	39.62	99.06	2.17
Inter-day (*n* = 25)						
0.5	0.47	94.83	10.48	0.46	92.97	10.06
1.5	1.45	96.46	7.92	1.47	97.85	5.91
15	15.20	101.30	8.07	15.44	102.91	5.81
40	39.58	98.95	8.81	40.88	102.21	5.46

Calc.: calculated; conc.: concentration.

**Table 3:** Extraction recovery of sirolimus and IS in porcine whole blood and lung tissue samples.

Concentration (ng/mL)	Extraction Recovery (%)	
	Whole Blood	Lung Tissue
Sirolimus		
1.5	59.73 ± 3.81	81.54 ± 6.32
15	56.80 ± 2.36	80.00 ± 1.47
40	63.33 ± 4.33	82.91 ± 4.76
Ascomycin (IS)		
1	102.43 ± 2.90	83.00 ± 2.63

**Table 4:** Matrix effect for sirolimus and IS in porcine whole blood and lung tissue samples.

Conc. (ng/mL)	Whole Blood			Lung Tissue		
	Absolute Matrix Effect (%) ^1^	Precision (CV, %) Set 1	Precision (CV, %) Set 2	Absolute Matrix Effect (%) ^1^	Precision (CV, %) Set 1	Precision (CV, %)Set 2
Sirolimus						
1.5	107.12	5.32	4.04	107.07	3.82	3.19
15	104.84	1.48	4.94	107.76	1.15	3.27
40	107.53	8.43	9.10	107.87	3.20	8.04
Ascomycin (IS)						
1	93.28	0.77	0.81	106.01	6.91	14.03

^1^ Absolute matrix effect was expressed as the ratio of mean peak area of the analyte added post deproteinization (set 1) to that of the neat standards (set 2) multiplied by 100. Conc.: concentration.

**Table 5:** Stability of sirolimus in porcine whole blood and lung tissue samples.

Storage Condition	Concentration (ng/mL)	Stability (%)	
		Whole Blood	Lung Tissue
Autosampler (24 h, 4 °C)	1.5	104.73 ± 5.44	100.12 ± 0.78
	15	95.63 ± 2.97	99.27 ± 1.67
	40	107.46 ± 12.39	101.13 ± 1.21
Freeze–thaw (3 cycles)	1.5	87.39 ± 1.36	105.75 ± 3.77
	15	85.41 ± 2.05	106.14 ± 4.82
	40	92.92 ± 5.62	106.51 ± 3.87
Short-term (4 h, 25 °C)	1.5	91.19 ± 0.52	96.67 ± 1.51
	15	93.96 ± 6.27	93.22 ± 4.17
	40	98.48 ± 2.35	92.44 ± 0.54
Long-term (3 months, −80 °C)	1.5	100.61 ± 4.31	106.24 ± 1.78
	15	94.62 ± 3.41	101.01 ± 5.05
	40	98.08 ± 4.94	101.92 ± 5.51

**Table 6:** Summary of pharmacokinetic parameters in whole blood for sirolimus (*n* = 24).

PK Parameters	Sirolimus
C_max_ (ng/mL)	7.79 ± 2.79
T_max_ (h)	2.26 ± 1.33
AUC_0–48 h_ (ng/mL·h) ^1^	156.9
AUC_inf_ (ng/mL·h) ^1^	164.8

^1^ Calculated based on the average sirolimus concentration in blood.

## Data Availability

The data presented in this study are available in the article and Supplementary Material.

## Supplementary Materials

The following are available online, Table S1: Selectivity of the LC-MS/MS method for sirolimus analysis in whole blood samples from six animals; Table S2: Selectivity of the LC-MS/MS method for sirolimus analysis in lung tissue samples from three animals; Table S3: Carry-over of sirolimus and IS in whole blood samples; Table S4: Carry-over of sirolimus and IS in lung tissue samples; Figure S1: Representative selected reaction monitoring (SRM) chromatograms of sirolimus (left) and IS (right) in carry-over study for blood samples; Figure S2: Representative selected reaction monitoring (SRM) chromatograms of sirolimus (left) and IS (right) in carry-over study for lung tissue samples.

## Sample Availability

Samples of the compound sirolimus are available from the authors.
