# Supplementary material for: Development and Validation of a Bioanalytical LC-MS/MS Method for Simultaneous Determination of Sirolimus in Porcine Whole Blood and Lung Tissue and Pharmacokinetic Application with Coronary Stents
Source: Molecules. 2021 Jan 15;26(2):425. doi: 10.3390/molecules26020425 (PMC7829871; doi:10.3390/molecules26020425)
Supplement: Supplementary file 1 [file molecules-26-00425-s001.pdf]

Supplementary Information

Development and Validation of a Bioanalytical LC-MS/MS Method for Simultaneous Determination of Sirolimus in Porcine Whole Blood and Lung Tissue and Pharmacokinetic Application with Coronary Stents

Thi-Thao-Linh Nguyen, Van-An Duong, Dang-Khoa Vo, Jeongae Jo, and Han-Joo Maeng

Table S1. Selectivity of the LC-MS/MS method for sirolimus analysis in whole blood samples fr om six animals.

Sample	Analyte peak area	Analyte RT (min)	IS peak area	IS RT (min)
Blank-1	N/A	N/A	N/A	N/A
Blank-2	N/A	N/A	N/A	N/A
Blank-3	N/A	N/A	N/A	N/A
Blank-4	N/A	N/A	N/A	N/A
Blank-5	N/A	N/A	N/A	N/A
Blank-6	N/A	N/A	N/A	N/A
IS 1 ng/mL-1	N/A	N/A	208281	5.33
IS 1 ng/mL-2	N/A	N/A	212089	5.34
IS 1 ng/mL-3	N/A	N/A	191517	5.33
IS 1 ng/mL-4	N/A	N/A	195272	5.33
IS 1 ng/mL-5	N/A	N/A	185359	5.33
IS 1 ng/mL-6	N/A	N/A	205676	5.32
LLOQ (0.5 ng/mL) + IS (1 ng/mL)-1	3789	5.49	216008	5.33
LLOQ (0.5 ng/mL) + IS (1 ng/mL)-2	3085	5.45	217314	5.32
LLOQ (0.5 ng/mL) + IS (1 ng/mL)-3	3380	5.46	210513	5.33
LLOQ (0.5 ng/mL) + IS (1 ng/mL)-4	3386	5.47	205627	5.32
LLOQ (0.5 ng/mL) + IS (1 ng/mL)-5	3912	5.47	210227	5.33
LLOQ (0.5 ng/mL) + IS (1 ng/mL)-6	3283	5.47	217035	5.32
PK sample at 5 min	27216	5.40	195702	5.33

N/A: not available

Table S2. Selectivity of the LC-MS/MS method for sirolimus analysis in lung tissue samples from three animals.

Sample	Analyte Peak Area	Analyte RT (min)	IS peak Area	IS RT (min)
Blank-1	N/A	N/A	N/A	N/A
Blank-2	N/A	N/A	N/A	N/A
Blank-3	N/A	N/A	N/A	N/A
IS 1 ng/mL-1	N/A	N/A	96006	5.35
IS 1 ng/mL-2	N/A	N/A	97311	5.35
IS 1 ng/mL-3	N/A	N/A	94506	5.35
LLOQ (0.5 ng/mL) + IS (1 ng/mL)-1	2265	5.43	90213	5.35
LLOQ (0.5 ng/mL) + IS (1 ng/mL)-2	2058	5.41	91134	5.35
LLOQ (0.5 ng/mL) + IS (1 ng/mL)-3	2023	5.40	94235	5.36
PK sample at 1h	18933	5.47	89693	5.36

N/A: not available

Table S3. Carry-over of sirolimus and IS in whole blood samples.

Sample	Repetition	Analyte peak area	Analyte RT (min)	IS peak area	IS RT (min)
Blood ULOQ 50 ng/mL		432102	5.44	213760	5.30
Blood Blank	1st	N/A	N/A	N/A	N/A
Blood LLOQ 0.5 ng/mL		3760	5.45	195667	5.31
Blood ULOQ 50 ng/mL		419706	5.45	219019	5.31
Blood Blank	2nd	N/A	N/A	N/A	N/A
Blood LLOQ 0.5 ng/mL		3853	5.44	205249	5.31
Blood ULOQ 50 ng/mL		442268	5.45	221647	5.30
Blood Blank	3rd	N/A	N/A	N/A	N/A
Blood LLOQ 0.5 ng/mL		4030	5.44	210326	5.30

N/A: not available

Table S4. Carry-over of sirolimus and IS in lung tissue samples.

Sample	Repetition	Analyte Peak area	Analyte RT (min)	IS Peak Area	IS RT (min)
Tissue ULOQ 50 ng/mL		283220	5.44	96881	5.31
Tissue Blank	1st	N/A	N/A	N/A	N/A
Tissue LLOQ 0.5 ng/mL		2283	5.44	105975	5.30
Tissue ULOQ 50 ng/mL		322277	5.45	97913	5.31
Tissue Blank	2nd	N/A	N/A	N/A	N/A
Tissue LLOQ 0.5 ng/mL		2832	5.45	107799	5.31
Tissue ULOQ 50 ng/mL		234822	5.44	84912	5.31
Tissue Blank	3rd	N/A	N/A	N/A	N/A
Tissue LLOQ 0.5 ng/mL		2750	5.43	107509	5.30

N/A: not available

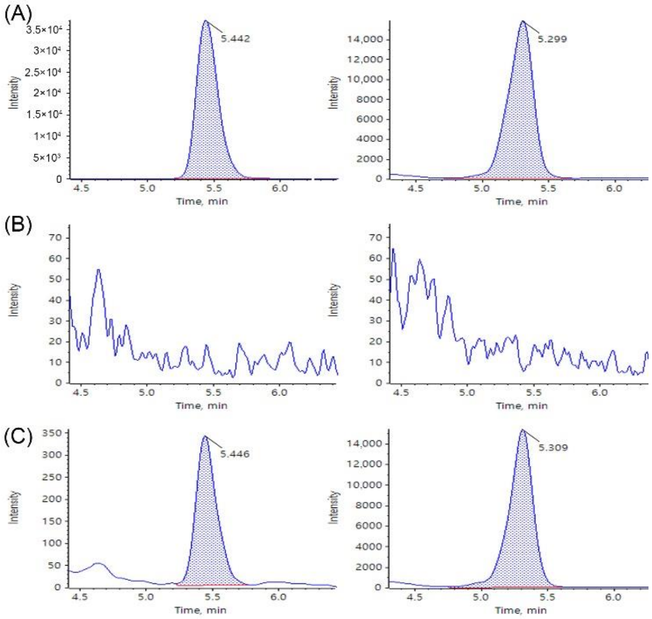

Figure S1. Representative selected reaction monitoring (SRM) chromatograms of sirolimus (**left**) and IS (**right**) in carry-over study for blood samples. (**A**) Blank porcine whole blood spiked with sirolimus at ULOQ 50 ng/mL and 1 ng/mL of IS. (**B**) Blank porcine whole blood. (**C**) Blank porcine whole blood spiked with sirolimus at LLOQ 0.5 ng/mL and 1 ng/mL of IS.

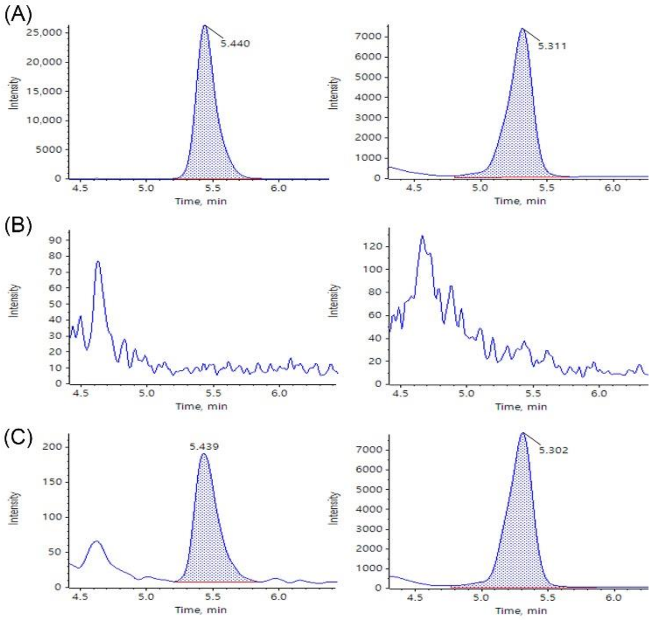

Figure S2. Representative selected reaction monitoring (SRM) chromatograms of sirolimus (**left**) and IS (**right**) in carry-over study for lung tissue samples. (**A**) Blank porcine lung tissue spiked with sirolimus at ULOQ 50 ng/mL and 1 ng/mL of IS. (**B**) Blank porcine lung tissue. (**C**) Blank porcine lung tissue spiked with sirolimus at LLOQ 0.5 ng/mL and 1 ng/mL of IS.
